# Identification and Screening of Lactate‐Related Genes as Molecular Markers for Early Diagnosis of Steroid‐Induced Osteonecrosis of the Femoral Head

**DOI:** 10.1096/fj.202504776R

**Published:** 2026-04-15

**Authors:** Zehua Wang, Shuhang Dong, Kaige Xu, Xinyu Tang, Sijia Guo, Yaping Jiang, Yingze Zhang, Tao Li

**Affiliations:** ^1^ Department of Joint Surgery The Affiliated Hospital of Qingdao University Qingdao China; ^2^ Department of Oral Implantology The Affiliated Hospital of Qingdao University Qingdao China; ^3^ Department of Orthopedics The Third Hospital of Hebei Medical University Shijiazhuang China

**Keywords:** immune microenvironment, infiltrating immunocytes, lactate‐related genes, machine learning algorithms, steroid‐induced osteonecrosis of the femoral head

## Abstract

Steroid‐induced osteonecrosis of the femoral head (SONFH) is a major cause of disability among young and middle‐aged adults. However, current diagnosis relies primarily on imaging findings and clinical manifestations, as stable and reliable molecular biomarkers for adjunctive diagnosis and risk stratification remain lacking, thereby hindering timely and effective intervention. Aberrant lactate metabolism is thought to contribute to the onset and progression of various inflammatory diseases by reshaping the inflammatory microenvironment and reprogramming immune responses. However, its role and regulatory mechanisms in SONFH remain understudied. In this study, we analyzed transcriptomic data from SONFH patients in the GEO database, integrating differential expression analysis with weighted gene co‐expression network analysis (WGCNA) to identify SONFH‐associated genes and co‐expression modules. Cross‐screening with lactate‐related genes (LRGs) curated in the MSigDB database yielded a set of LRGs closely associated with SONFH. Unsupervised consensus clustering was then applied to stratify patients into molecular subtypes, and a machine–learning–based diagnostic model was constructed. In parallel, gene set variation analysis (GSVA) and CIBERSORT were used to characterize metabolic states and immune cell infiltration across subtypes, with a focus on LRGs implicated in metabolic reprogramming and immune dysregulation. Finally, bone marrow–derived mesenchymal stem cells (BMSCs) were collected from Sprague–Dawley rats and humans, along with peripheral blood from patients, and in vitro experiments confirmed significant downregulation of BPGM, FBXL4, and RHAG in SONFH, genes closely linked to bone metabolic imbalance and immune microenvironment remodeling. Collectively, these findings systematically elucidate the potential molecular regulatory role of LRGs in SONFH and provide a theoretical basis for its auxiliary diagnosis and the development of targeted therapeutic strategies.

## Graphical abstract

Identification and validation of lactate‐related genes biomarkers for steroid‐induced osteonecrosis of the femoral head through integrated bioinformatics analysis, machine learning, and experimental verification.

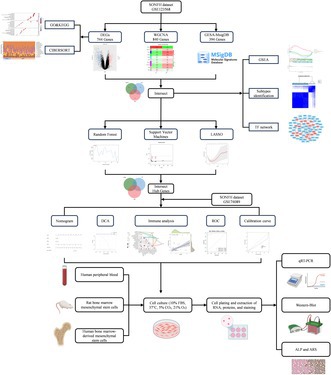

## 1 Introduction

Osteonecrosis of the femoral head (ONFH) is a progressive osteonecrotic disorder caused by impaired blood supply to the femoral head. It predominantly affects young and middle‐aged adults and is a common cause of hip pain and functional limitation [1, 2, 3]. The onset and progression of ONFH involve multiple pathological mechanisms, including impaired microcirculatory perfusion [4], lipid metabolism disorders [5], osteoblast apoptosis, and disruption of local metabolic homeostasis [3, 6]. Etiologically, ONFH can be classified into traumatic and non‐traumatic types. Common precipitating factors include glucocorticoid use, chronic alcohol consumption, and prior trauma, among which steroid exposure is the most prevalent non‐traumatic risk factor [7].

Steroid‐induced osteonecrosis of the femoral head (SONFH) is a non‐traumatic osteonecrosis caused by prolonged or high‐dose glucocorticoid exposure, which precipitates compromised blood supply to the femoral head, osteocyte necrosis, and structural collapse of the bone [8]. Glucocorticoids (GCs) are regarded as one of the principal pathogenic factors driving the onset and progression of SONFH [9]. Extensive evidence shows that prolonged excessive GC exposure dysregulates lipid metabolism, induces hypertrophy of intramedullary adipocytes, injures vascular endothelial cells, and inhibits angiogenesis; together, these changes produce a sustained reduction in microcirculatory perfusion of the femoral head and generate a profoundly ischemic and hypoxic local microenvironment [10, 11, 12, 13]. In this stressed state, osteocytes and bone marrow mesenchymal stem cells (BMSCs) undergo metabolic reprogramming, shifting energy production from oxidative phosphorylation to glycolysis, which leads to substantial local lactate accumulation [14]. Excess lactate not only reflects hypoxia‐induced metabolic remodeling but may also regulate key pathological processes—including vascular repair, inflammatory immune responses, and bone metabolic homeostasis—by altering local acid–base balance and modulating lactate production, transport, and utilization. Notably, lactate is not merely the end product of glycolysis; it also functions as a signaling molecule. Through lactylation, a recently described post‐translational modification, lactate can modify histones and non‐histone proteins, thereby influencing chromatin organization, transcriptional regulation, inflammatory gene expression, and cellular differentiation [15, 16]. Given that SONFH is characterized by persistent ischemia, bone metabolic imbalance, a dysregulated inflammatory microenvironment, and abnormal osteogenic–adipogenic differentiation [17], lactate‐related genes (LRGs) may play a central role in regulating these pathological processes through metabolic reprogramming and maintenance of microenvironmental homeostasis. These functions may involve multiple aspects of SONFH pathogenesis, including osteoblast activity, the differentiation fate of BMSCs, angiogenesis, and local immune homeostasis. However, despite increasing recognition of the biological significance of LRGs in ischemic tissue injury, inflammatory disease, and tissue repair, systematic investigations of their expression patterns, regulatory networks, key targets, and molecular mechanisms in SONFH remain limited.

Accordingly, we performed a systematic bioinformatics analysis of RNA‐sequencing data from SONFH patients retrieved from the Gene Expression Omnibus (GEO). Differentially expressed genes (DEGs) between hormone‐treated unaffected individuals and SONFH patients were first identified. We then integrated weighted gene co‐expression network analysis (WGCNA) with LRGs curated from the Molecular Signatures Database (MSigDB) to select LRGs significantly associated with SONFH. Using consensus unsupervised clustering on the expression profiles of these LRGs, patients were stratified into molecular subtypes to explore inter‐subtype biological differences. Next, Support Vector Machine–Recursive Feature Elimination (SVM‐RFE), Least Absolute Shrinkage and Selection Operator (LASSO) regression, and Random Forest (RF) algorithms were applied to comprehensively identify key LRGs. A predictive model was then developed and evaluated for its diagnostic performance as a potential SONFH biomarker panel. Finally, bone marrow‐derived mesenchymal stem cells (BMSCs) and peripheral blood samples were collected from patients with avascular necrosis of the femoral head and femoral neck fractures, followed by analysis using qRT‐PCR and western blotting. This study aims to clarify the molecular regulatory role of LRGs in SONFH and to provide a theoretical basis for auxiliary diagnosis and targeted intervention.

## 2 Materials and Methods

### 2.1 Data Collection

We retrieved the GSE123568 dataset (platform GPL15207) and the GSE74089 dataset (platform GPL13497) from the GEO database (https://www.ncbi.nlm.nih.gov/geo/). GSE123568 includes peripheral blood samples from 30 patients with SONFH and 10 steroid‐treated unaffected individuals and was subjected to transcriptomic analysis to assess differential gene expression [18]. GSE74089 includes four healthy hip cartilage samples and four hip cartilage samples from patients with avascular necrosis of the femoral head [19]. This independent cohort was used to validate the expression patterns of candidate genes across distinct sample types. In addition, we obtained a set of 394 LRGs from MsigDB (https://www.gsea‐msigdb.org/gsea/msigdb/) [20].

### 2.2 Analysis and Functional Annotation of Differentially Expressed Genes

We used the limma package for data normalization and differential expression analysis to identify DEGs between SONFH patients and unaffected individuals in the GSE123568 dataset [21]. DEGs were defined using thresholds of |log_2_FC| > 0.75 and adjusted *p* < 0.05. The results were visualized with the pheatmap and ggplot2 packages.

Subsequently, we performed Gene Ontology (GO) enrichment analysis and Kyoto Encyclopedia of Genes and Genomes (KEGG) pathway analysis on the DEGs using the clusterProfiler package to elucidate functional differences between SONFH patients and unaffected individuals [22]. The org. Hs. eg.db annotation database was used as the reference gene set, and statistical significance was defined as *p* < 0.05. Enrichment terms with *p*‐values < 0.05 were considered statistically significant.

### 2.3 Weighted Gene Co‐Expression Network Analysis

We conducted WGCNA on the GSE123568 dataset using the WGCNA package [23]. Genes with variance greater than 25% were selected, and Pearson correlation coefficients were calculated to construct an adjacency matrix. The soft‐thresholding power was set to 8, and the resulting adjacency matrix was subsequently transformed into a topological overlap matrix (TOM). Gene modules with similar expression patterns were identified using the dynamic tree‐cutting algorithm. Key modules were determined by evaluating the correlation between module eigengenes (MEs) and the SONFH phenotype. Gene Significance (GS) and Module Membership (MM) were then calculated to assess the correlations of individual genes with the SONFH phenotype and the corresponding module eigengenes, respectively. Genes with GS > 0.5 and MM > 0.5 were defined as SONFH‐associated characteristic genes. Finally, these genes were intersected with DEGs and LRGs to identify LRGs significantly associated with SONFH.

### 2.4 Gene Enrichment Analysis and Transcription Factor Network Analysis

We conducted gene set enrichment analysis (GSEA) using the limma and clusterProfiler packages to identify potential KEGG pathways associated with the biomarkers, defining statistical significance as *p* < 0.05 [24]. In addition, transcription factors (TFs) regulating the hub genes were predicted using the JASPAR transcription factor binding database (https://jaspar.elixir.no/about/), and a regulatory network was visualized in Cytoscape [25].

### 2.5 Consensus Cluster Analysis and Differences Between Subtypes

We identified molecular subtypes of SONFH by analyzing the expression profiles of SONFH‐associated LRGs. Unsupervised consensus clustering was performed using the ConsensusClusterPlus package [26]. To ensure clustering stability, the k‐means algorithm was applied with 1000 iterations, a sample consistency cutoff of 0.8, and a feature consistency cutoff of 1. We then compared immune microenvironment differences between the two subtypes and conducted gene set variation analysis (GSVA) to explore subtype‐specific functional variation [27]. Finally, differentially expressed genes between the subtypes were calculated using the limma package, and GO and KEGG enrichment analyses were performed to characterize their functional distinctions.

### 2.6 Machine Learning Screening of Key Genes and Construction of Diagnostic Models

We employed SVM‐RFE, LASSO regression, and RF algorithms to further identify key genes within the GSE123568 dataset. Genes jointly selected by all three methods were designated as key genes [28, 29, 30]. Because nomograms are widely used in clinical research to estimate the probability of clinical outcomes, we constructed a nomogram incorporating these key genes using the rms package to predict the risk of SONFH. To validate their predictive performance, we used GSE123568 and GSE74089 as the training and validation sets, respectively. The discriminatory ability of these key genes was assessed by constructing receiver operating characteristic (ROC) curves using the pROC package. To improve the robustness of the results under small‐sample conditions, we further estimated the area under the curve (AUC), 95% confidence interval (CI), and standard deviation (SD) for each candidate gene using 1000 bootstrap resamples. Calibration curves were generated to evaluate model calibration, and decision curve analysis (DCA) was performed to assess clinical utility [31].

### 2.7 Analysis of Immune Cell Infiltration

We applied the CIBERSORT algorithm to estimate the relative abundances of 22 immune cell types in the SONFH microenvironment. Spearman correlation analysis was then performed using the ggExtra package to assess associations between the biomarkers and differentially abundant immune cells [32, 33]. The immune infiltration results were visualized with the ggplot2 package.

### 2.8 Isolation of Rat Bone Marrow Mesenchymal Stem Cells

Three‐week‐old SD rats were anesthetized with isoflurane inhalation (3%–4% for induction and 1.5%–2.5% for maintenance in oxygen) and euthanized by isoflurane overdose followed by cervical dislocation, in accordance with institutional animal care guidelines. The carcasses were then immersed in 75% ethanol for 15 min for disinfection. Bilateral lower limbs were dissected and transferred to PBS containing 1% penicillin–streptomycin for temporary storage. Under a laminar flow hood, muscle tissue was removed from the limbs, the epiphyses at both ends of the femur and tibia were excised, and the medullary cavities were repeatedly flushed with α‐MEM containing 1% penicillin–streptomycin to obtain a cell suspension. The suspension was centrifuged, the supernatant was discarded, and the cells were resuspended in α‐MEM complete medium supplemented with 10% FBS and seeded into culture flasks. Cultures were incubated at 37°C in a humidified incubator with 5% CO_2_. The medium was first changed after 3 days to remove non‐adherent cells. Typical fibroblast‐like colonies were observed by day 5. Cells were then passaged for expansion, and third‐passage (P3) cells were used in subsequent experiments.

### 2.9 Isolation of Human Bone Marrow Mesenchymal Stem Cells and Peripheral Blood

This study was conducted in accordance with the Declaration of Helsinki and was approved by the Ethics Committee of Qingdao University Affiliated Hospital. Bone marrow tissue and peripheral blood were collected from three patients with SONFH as the experimental group and from three patients with femoral neck fractures as the control group. All patients underwent total hip arthroplasty (THA) at Qingdao University Affiliated Hospital. All participants were fully informed about the study procedures and provided written informed consent.

Peripheral venous blood samples were collected on the second day of hospitalization into EDTA‐anticoagulated tubes. Immediately after collection, the tubes were gently inverted to ensure adequate mixing and then centrifuged to separate plasma from cellular components for subsequent Quantitative real‐time PCR (qRT‐PCR) analysis.

During total hip arthroplasty in patients with SONFH, approximately 5 mL of bone marrow was aspirated from the femoral medullary cavity. The sample was diluted 1:1 with PBS and thoroughly mixed. Percoll was then slowly added along the inner wall of the centrifuge tube to establish a density gradient, followed by centrifugation at 2000 rpm for 25 min. The mononuclear cell layer at the white interface was carefully aspirated and centrifuged in PBS at 1000 rpm for 5 min to remove impurities and residual blood components; this washing step was repeated three times. The resulting cell pellet was collected and resuspended in α‐MEM complete medium supplemented with 10% FBS. After 3 days, the medium was replaced to remove non‐adherent cells. Cell morphology and growth were monitored, and when cultures reached approximately 90% confluence, cells were digested with trypsin, resuspended, and passaged at a 1:2 ratio. P3 cells were used in subsequent experiments.

### 2.10 Osteogenic Induction and Staining Observation

BMSCs were pretreated with dexamethasone at a concentration previously reported in the literature (10 μM) [34], followed by osteogenic induction. For osteogenic induction, 1 × 10^5^ cells were seeded per well in 12‐well plates. When cells reached 60%–70% confluence, the medium was replaced with osteogenic induction medium (OM), consisting of α‐MEM, 10% FBS, 100 nM dexamethasone (Sigma‐Aldrich), 10 mM β‐glycerophosphate (Sigma‐Aldrich), and 50 μg/mL L‐ascorbic acid (Sigma‐Aldrich). The medium was changed every 2 days. For alkaline phosphatase (ALP) staining, cells were cultured in OM for 7 days, fixed with 4% paraformaldehyde (PFA) for 20 min, and then incubated with BCIP/NBT solution at room temperature in the dark for 30 min according to the ALP activity detection kit instructions. Cells were washed twice with distilled water and examined under a microscope. For alizarin red S (ARS) staining, cells were cultured in OM for 14 days, fixed with 4% PFA for 20 min, and then incubated with ARS at room temperature in the dark for 30 min.

### 2.11 RT‐qPCR Detection

In this study, total RNA was extracted from BMSCs using TRIzol reagent and reverse‐transcribed into complementary DNA (cDNA) with a commercial reverse transcription kit. Quantitative real‐time PCR was performed using a SYBR Green–based kit, and amplification was monitored by measuring fluorescence intensity. All primer sequences are provided in Table 1.

**TABLE 1:** Primer sequences of qRT‐PCR.

Gene	Species	Forward primer (5′‐3′)	Reverse primer (5′‐3′)
FBXL4	Human	AGAGGACGCCACCTAATTTTCA	GGATTTGCAGAACAAGCGAGAAT
BPGM	Human	TGCTTGGAATAAGGAGAACCGT	CCACAGTTCCGAGCTTCCTC
RHAG	Human	TATGAAACGGACCAGACTGTTCT	CAGCAACGAGTAGGTTGATACC
FBXL4	Mouse	CCTAGTGCTTCCTTGCCATTC	ACTTGCTGCTCAAAAGTCAGT
BPGM	Mouse	CTGAATGAGCGTCACTATGGAG	GGGGTCACGTTGTAGCTTCT
RHAG	Mouse	CCTTTGGAGCTTACTTTGGCT	TCATTTGGGTGTTCACATCTGAG

### 2.12 Western Blot

In this study, BMSCs were cultured in 6‐well plates and, after treatment, lysed with radioimmunoprecipitation assay (RIPA) buffer supplemented with protease inhibitors to extract total protein. Protein concentrations were determined using a BCA assay kit. Equal amounts of protein were separated on 4%–20% sodium dodecyl sulfate–polyacrylamide gels (SDS‐PAGE) and transferred to polyvinylidene fluoride (PVDF) membranes. After blocking for 2 h in Tris‐buffered saline with Tween (TBST) containing 5% skim milk, the membranes were incubated with the following primary antibodies (Wuhan Proteintech): rabbit monoclonal anti‐GAPDH (10494‐1‐AP, 1:20 000), rabbit polyclonal anti‐HSP70 (10995‐1‐AP, 1:25 000), rabbit polyclonal anti‐RUNX2 (20700‐1‐AP, 1:5000), rabbit polyclonal anti‐FBXL4 (24754‐1‐AP, 1:10 000), rabbit monoclonal anti‐RHAG (67714‐1‐Ig, 1:2000), and rabbit polyclonal anti‐BPGM (17173‐1‐AP, 1:4000). Membranes were incubated with primary antibodies overnight at 4°C on a rocking platform. After three washes with TBST, goat anti‐rabbit IgG (H + L) secondary antibody (SA00001‐2, Proteintech, Wuhan) was added and incubated at room temperature for 1 h. Protein bands were visualized using Feimin ECL chemiluminescent reagent (SB‐WB004, Share‐Bio) and detected with an enhanced chemiluminescence imaging system. Band grayscale values were quantified using ImageJ, and relative protein expression levels were calculated by normalizing target band intensities to those of the internal reference. All experiments were performed in triplicate.

### 2.13 Statistical Analysis

All statistical analyses were performed using R (version 4.3.3). Data were first assessed for normality and were found to be approximately normally distributed. Differences between the two groups were analyzed using unpaired *t*‐tests, whereas comparisons among more than two groups were performed using one‐way analysis of variance (ANOVA). Statistical significance was defined as *p < 0.05, **p < 0.01, and ***p < 0.001.

## 3 Result

### 3.1 Expression and Functional Analysis of Differentially Expressed Genes in SONFH Patients

**FIGURE 1:**
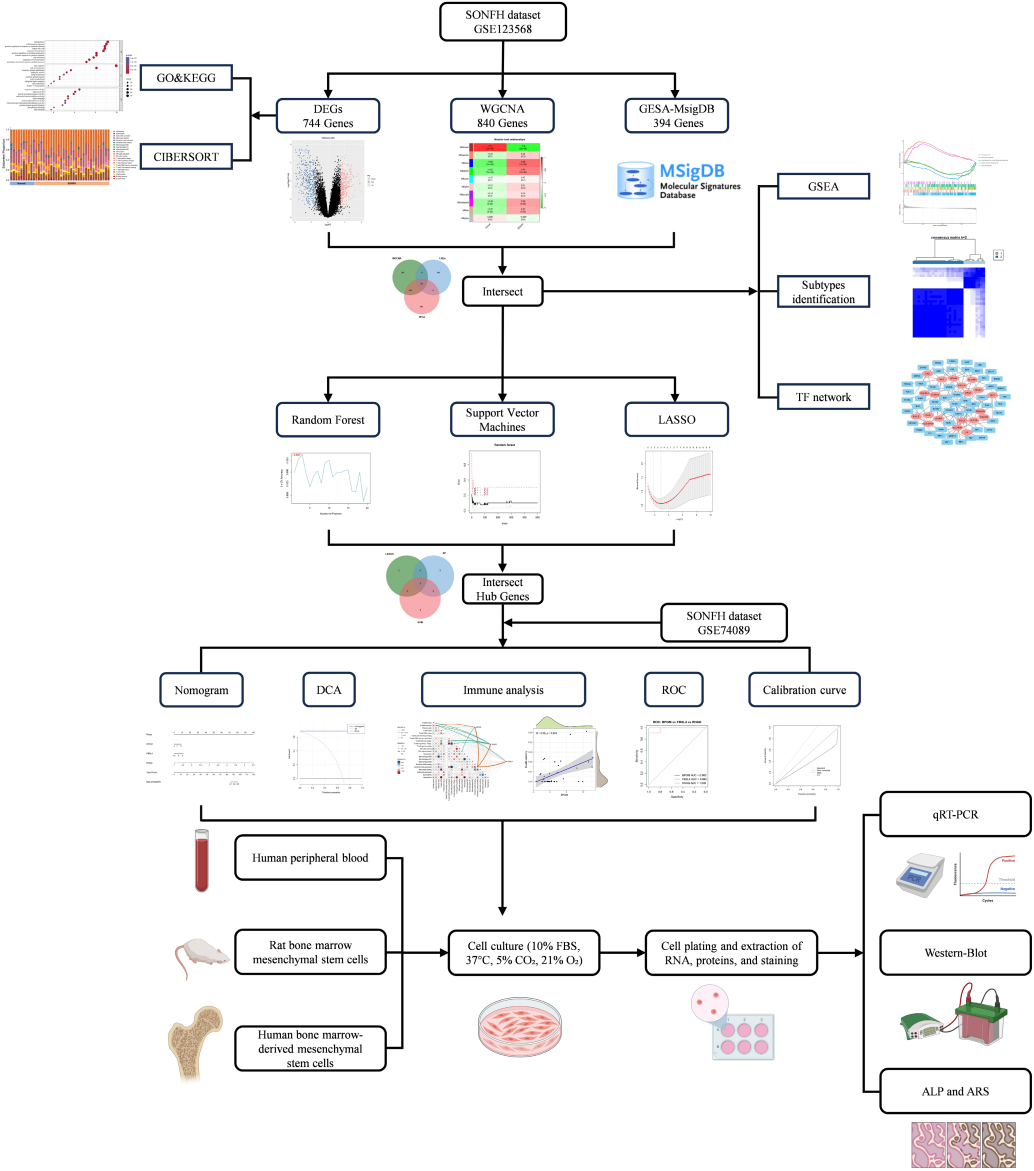
Flowchart of the study design.

**FIGURE 2:**
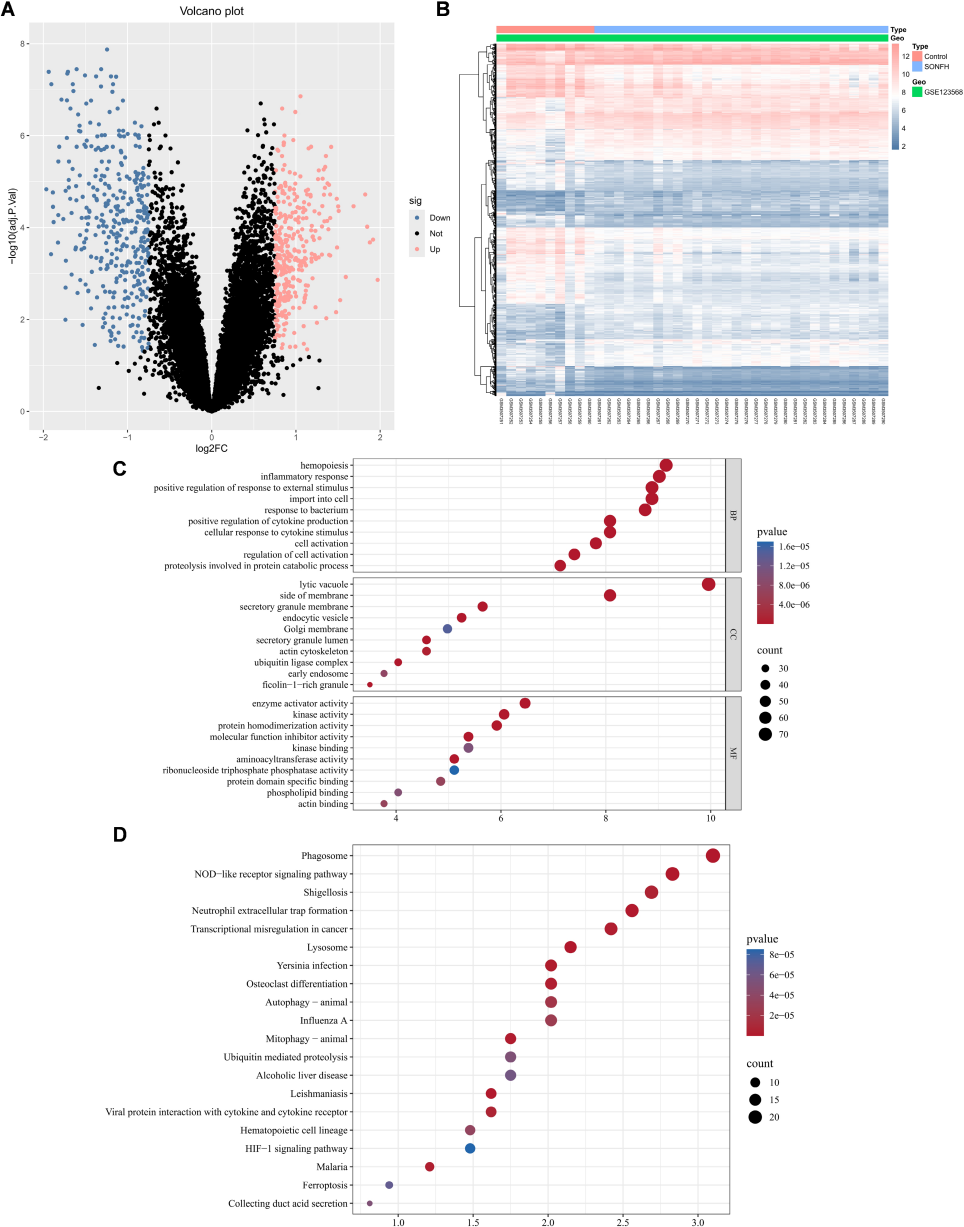
Screening and analysis of DE associated with SONFH. (A) Volcano plot of differentially expressed genes. (B) Heatmap of differentially expressed genes. (C) Top 10 biological processes, cellular components, and molecular functions in GO analysis. (D) Top 20 pathways in KEGG analysis.

The workflow of this study is illustrated in Figure 1. Using thresholds of |log_2_FC| > 0.75 and adjusted *p* < 0.05, we identified 744 differentially expressed genes (DEGs) from the GSE dataset, including 338 upregulated and 406 downregulated genes. These DEGs were visualized with a heatmap and a volcano plot (Figure 2A,B). GO and KEGG enrichment analyses were then performed on the 744 DEGs. GO biological process (BP) terms were predominantly related to immune and inflammatory responses, such as hemostasis, inflammatory response, and response to external stimuli. Cellular component (CC) terms were enriched in intracellular vesicular structures, including lysosomes, secretory granule membranes, and endocytic vesicles. Molecular function (MF) terms were mainly enriched in enzyme activation, kinase activity, and regulation of molecular function. KEGG pathway analysis indicated that these DEGs were closely associated with pathways involved in phagosome formation, neutrophil extracellular trap formation, autophagy, mitophagy, ferroptosis, and osteoclast differentiation related to bone resorption (Figure 2C,D).

### 3.2 Construction of WGCNA and Identification of Key Module Genes Related to SONFH

**FIGURE 3:**
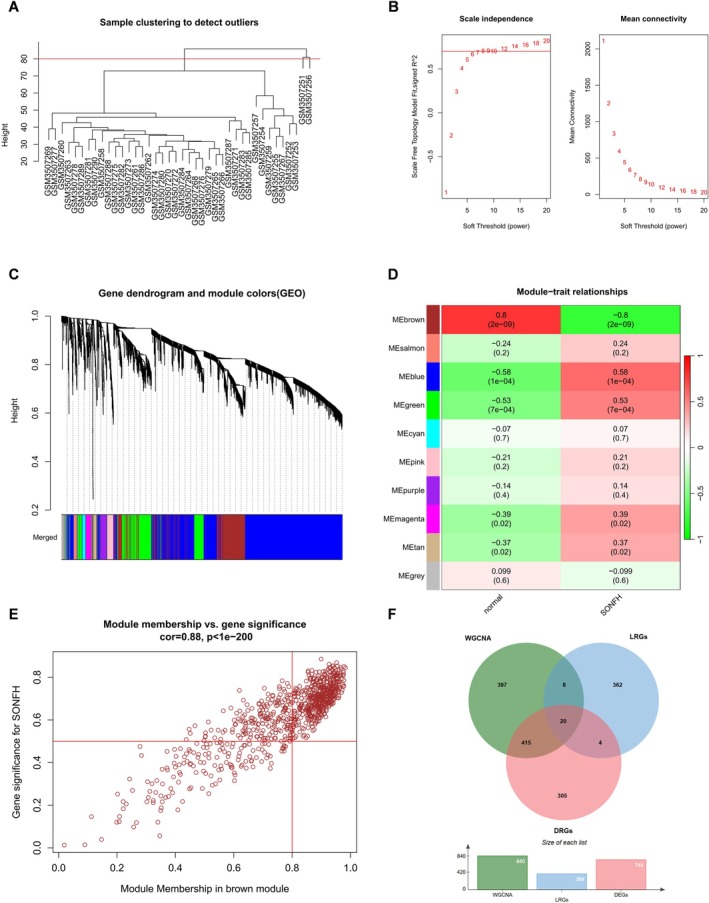
WGCNA Analysis. (A–D) WGCNA analysis revealed one module most strongly associated with SONFH: The MEbrown module showed a positive correlation with SONFH. (E) Genes with correlation coefficients greater than 0.5 in the MEbrown module. (F) 20 SONFH‐associated lactate‐related genes.

**FIGURE 4:**
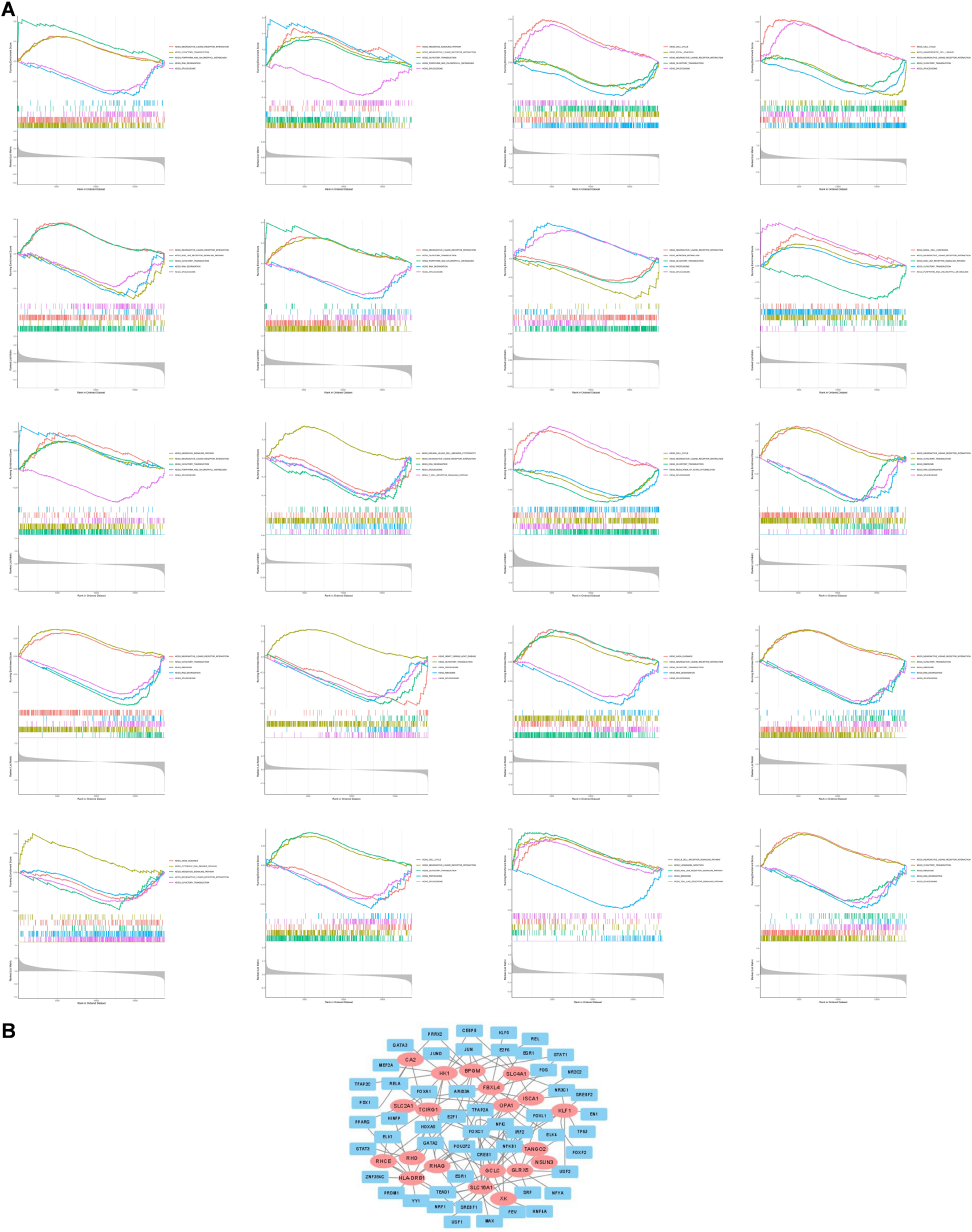
Results of gene enrichment analysis and transcription factor network analysis.

To further identify key genes associated with SONFH, we performed WGCNA on the top 25% most variable genes (Figure 3) to construct co‐expression modules related to SONFH. A soft‐thresholding power of 8 was selected as the optimal value, yielding a scale‐free topology fit index (*R* ^2^) of 0.85 and an average connectivity close to zero, indicating that this parameter adequately satisfied scale‐free network characteristics. The resulting TOM was then used to identify gene modules correlated with clinical traits. The brown module (correlation coefficient = 0.8, *p* = 2 × 10^−9^) showed the strongest association with SONFH (Figure 3A–E). By intersecting the 840 genes in this module (with significance and correlation coefficients > 0.5) with the 744 DEGs and 394 LRGs, we identified 20 lactate‐related differentially expressed genes (LR‐DEGs) in SONFH: ISCA1, SLC2A1, HK1, SLC16A1, BPGM, GLRX5, SLC4A1, XK, FBXL4, GCLC, RHAG, KLF1, CA2, TANGO2, RHD, OPA1, RHCE, NSUN3, TCIRG1, and HLA‐DRB1 (Figure 3F). In addition, GSEA and TF analysis were performed on these genes to investigate their functional roles and regulatory mechanisms in SONFH (Figure 4).

### 3.3 Establishment of Subtypes Based on Key Genes and Functional Analysis

**FIGURE 5:**
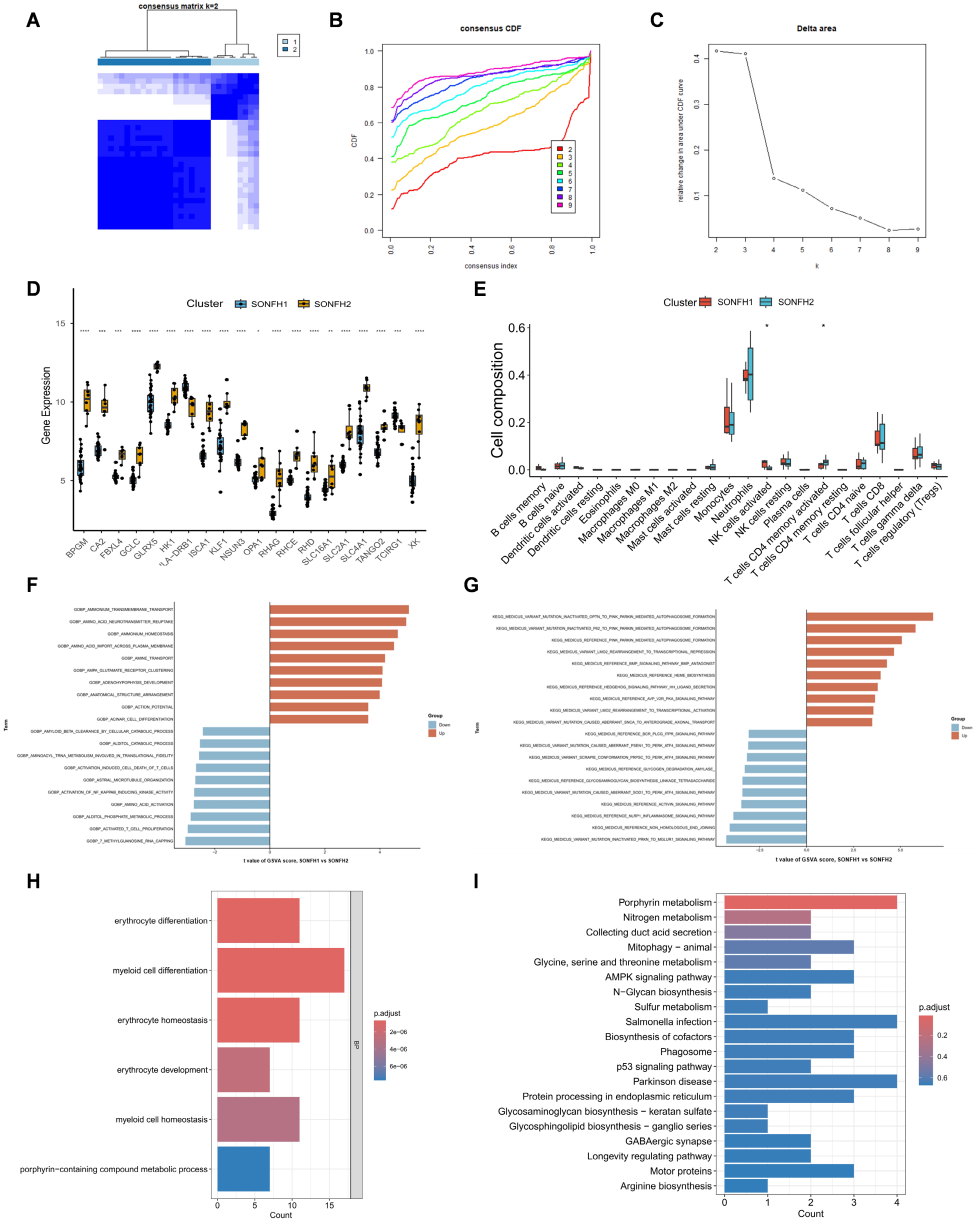
Subtype analysis of SONFH based on lactate‐related genes. (A–C) Consensus clustering analysis identified two distinct subtypes of SONFH. (D) Expression patterns of 20 genes in two SONFH subtypes. (E) Differences in immune cell infiltration between two SONFH subtypes. (F, G) GSVA analysis results for the two SONFH subtypes. (H) GO analysis of differentially expressed genes between two SONFH subtypes. (I) KEGG pathway enrichment analysis of differentially expressed genes between two SONFH subtypes.

We performed unsupervised clustering of SONFH patients based on LR‐DEG expression profiles and identified two molecular subtypes, SONFH1 and SONFH2 (Figure 5A–C). In SONFH1, SLC2A1, SLC4A1, TANGO2, and XK were upregulated, whereas HLA‐DRB1 and TCIRG1 were upregulated in SONFH2 (Figure 5D). Immune infiltration analysis revealed that activated NK cells were significantly elevated in SONFH1, while activated CD4^+^ memory T cells were significantly increased in SONFH2 (Figure 5E). GSVA analysis showed that, compared with SONFH2, SONFH1 exhibited marked downregulation of pathways related to metabolism and cellular transport. Still, upregulation of pathways involved in autophagy, stress responses, and cellular signaling regulation, indicating distinct biological functional characteristics between the two subtypes (Figures 5F,G).

To further characterize the differences between SONFH1 and SONFH2, we compared gene expression profiles between the two groups. Using thresholds of |log_2_FC| > 0.75 and adjusted *p* < 0.05, we identified 120 differentially expressed genes. GO and KEGG enrichment analyses were then performed on these genes to elucidate their potential biological functions. GO enrichment analysis indicated that these genes were primarily involved in erythroid and myeloid cell differentiation, maintenance of cellular homeostasis, and porphyrin compound metabolism, suggesting close links to hematopoiesis and tissue metabolic regulation. Similarly, KEGG enrichment analysis showed that these genes were predominantly enriched in pathways related to porphyrin and nitrogen metabolism, mitochondrial autophagy, amino acid metabolism, AMPK signaling, and lysosomal and phagosomal pathways, all of which are closely associated with energy metabolism remodeling and cellular stress responses (Figure 5H,I).

### 3.4 Development and Validation of SONFH Biomarkers Based on LR‐DEG

**FIGURE 6:**
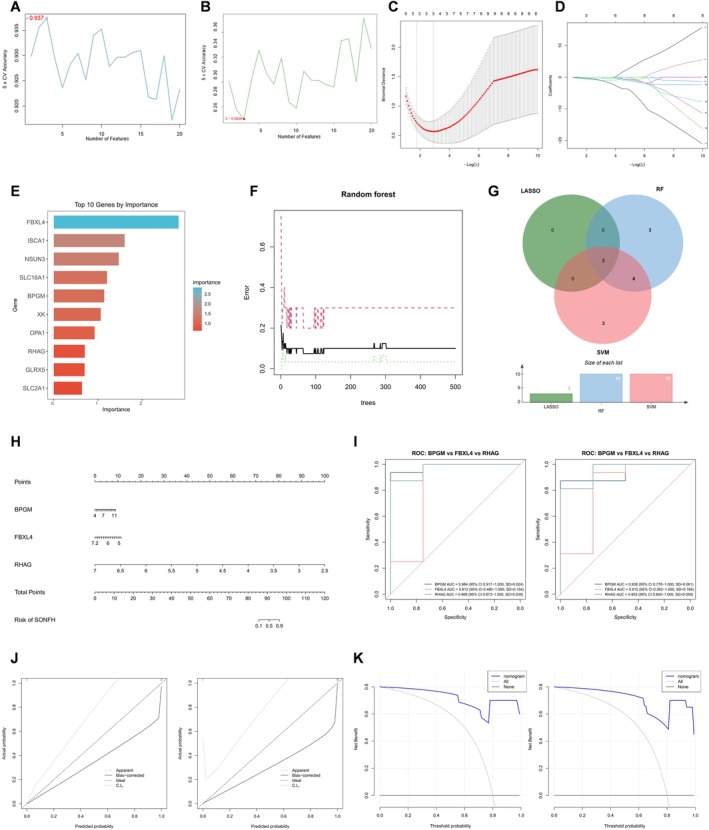
Screening of lactate‐related biomarkers for SONFH. (A, B) Screening core genes in LR‐DEGs using the SVM algorithm. (C, D) Screening core genes in LR‐DEGs using the LASSO algorithm. (E, F) Screening core genes in LR‐DEGs using the RF algorithm. (G) Three key LR‐DEGs were identified through the intersection of results from three machine learning algorithms. (H) Nomogram for predicting the risk of SONFH. (I–K) Validate the predictive accuracy and clinical utility of this nomogram using ROC curves, calibration curves, and DCA curves.

To identify key genes, we first applied SVM‐RFE to the 20 LR‐DEGs and obtained 10 feature genes: HK1, TANGO2, RHAG, OPA1, SLC16A1, BPGM, FBXL4, NSUN3, TCIRG1, and ISCA1 (Figure 6A,B). We then analyzed these genes using the LASSO algorithm, which selected three feature genes: BPGM, FBXL4, and RHAG (Figure 6C,D). In addition, the RF algorithm identified 10 feature genes: FBXL4, ISCA1, NSUN3, SLC16A1, BPGM, XK, OPA1, RHAG, GLRX5, and SLC2A1 (Figure 6E,F). Intersecting the genes selected by all three algorithms yielded three core genes: BPGM, FBXL4, and RHAG (Figure 6G). Relevant information for these three hub genes is summarized in Table 2.

**TABLE 2:** Hub genes and their biological function.

Gene symbol	Description	Biological function
FBXL4	F‐box and leucine‐rich repeat protein 4	SCF ubiquitin ligase subunit
BPGM	Bisphosphoglycerate mutase	2,3‐DPG metabolic enzyme
RHAG	Rh‐associated glycoprotein	Erythrocyte ammonium and CO_2_ transporter

Subsequently, a nomogram incorporating these three genes was constructed to predict SONFH risk (Figure 6H), with each gene assigned an individual score. The predicted probability of SONFH was obtained by summing the scores for the three genes. We then performed ROC analyses for the three core genes in both the training and validation sets. In the training set, the AUC was 0.984 for BPGM (bootstrap 95% CI: 0.917–1.000; SD = 0.024), 0.812 for FBXL4 (bootstrap 95% CI: 0.480–1.000; SD = 0.154), and 0.969 for RHAG (bootstrap 95% CI: 0.873–1.000; SD = 0.039). In the validation set, the AUC was 0.938 for BPGM (bootstrap 95% CI: 0.778–1.000; SD = 0.024), 0.812 for FBXL4 (bootstrap 95% CI: 0.393–1.000; SD = 0.168), and 0.953 for RHAG (bootstrap 95% CI: 0.800–1.000; SD = 0.059). These results indicate strong discriminatory performance of the model (Figure 6I). Calibration curves were generated to evaluate agreement between predicted and observed probabilities, and the curves closely followed the diagonal reference line, indicating high predictive accuracy (Figure 6J). The clinical utility of the nomogram was further assessed using DCA, which showed a high net benefit across a wide range of threshold probabilities in both the training and validation cohorts, supporting the nomogram as a useful tool for SONFH risk prediction (Figure 6K).

### 3.5 Immune Infiltration and Interactions Between Core Genes and Immune Cells

**FIGURE 7:**
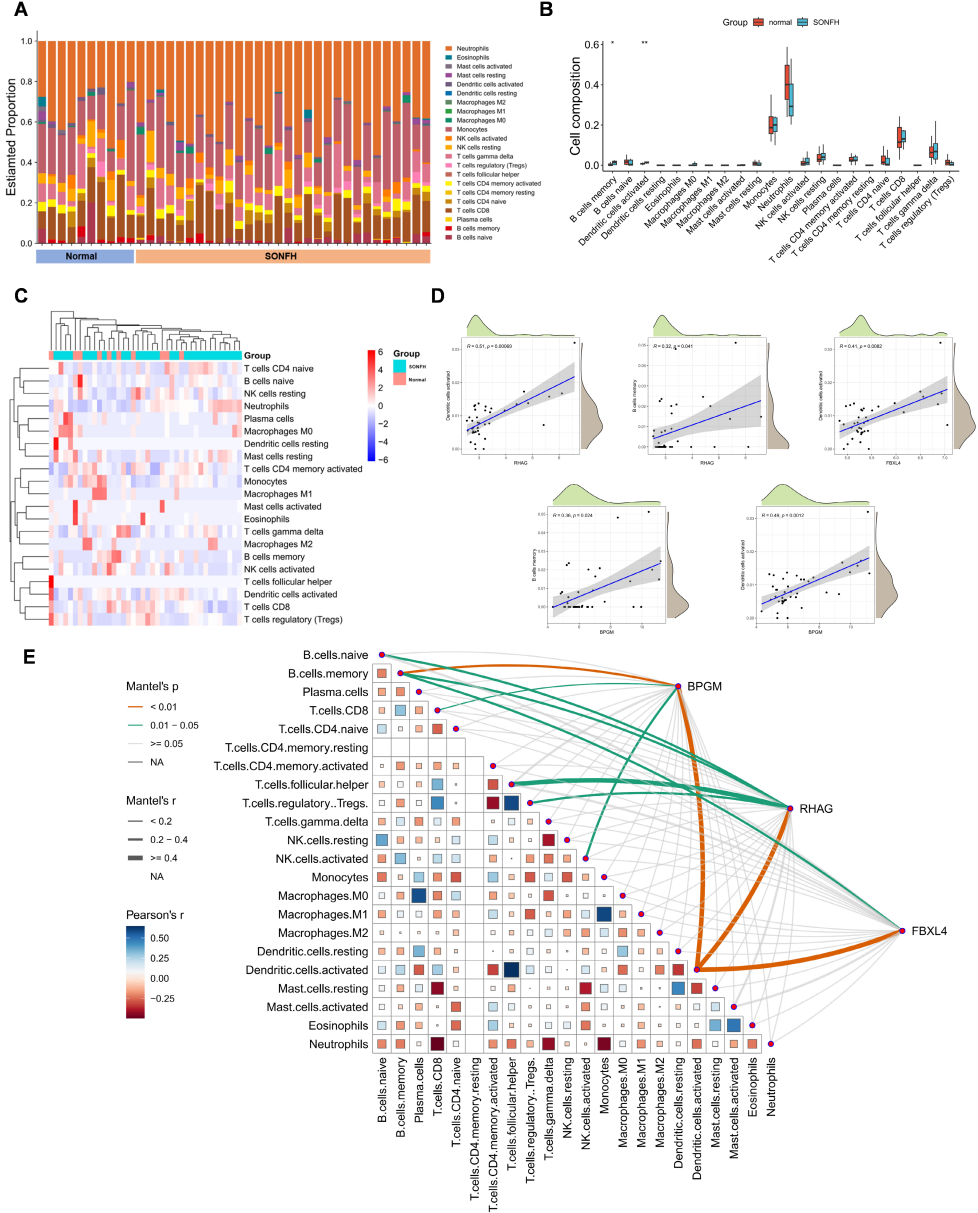
Immune infiltration analysis of DEGs and lactation‐related genes in SONFH. (A) Determine the relative proportion of infiltrating immune cells in SONFH using the CIBERSORT algorithm. (B) Differential analysis of abundance in 22 immune cell types in SONFH samples. (C) Heatmap of the relative abundance of 22 immune cell types in experimental and normal samples. (D, E) Pearson correlation analysis between three key genes and immune cells.

We analyzed immune cell composition in the GSE123568 dataset using the CIBERSORT algorithm. Compared with the unaffected control group, the SONFH group exhibited a distinct immune cell profile, characterized by higher proportions of memory B cells and activated dendritic cells (Figure 7A–C).

Correlation analyses showed that expression of the core genes was significantly associated with infiltration of multiple immune cell subsets. RHAG expression was positively correlated with activated dendritic cells (*R* = 0.51, *p* = 0.00069) and memory B cells (*R* = 0.32, *p* = 0.041). FBXL4 expression was also positively correlated with activated dendritic cells (*R* = 0.41, *p* = 0.0082). BPGM expression was positively correlated with memory B cells (*R* = 0.36, *p* = 0.024) and with activated dendritic cell infiltration (*R* = 0.49, *p* = 0.0012) (Figure 7D). Taken together, these findings suggest that the core genes may contribute to reshaping the SONFH immune microenvironment by modulating the infiltration and function of dendritic cells and B cells. Correlation network analysis further revealed significant associations between BPGM, RHAG, and FBXL4 and multiple immune cell infiltration features: BPGM and RHAG were positively correlated with activated dendritic cells, monocytes, and memory B cells, whereas FBXL4 was strongly associated with dendritic cells, M1 macrophages, and activated CD4^+^ T cells. Mantel tests further confirmed the overall association structure between these genes and immune infiltration patterns, supporting a pivotal role of LRGs in regulating the SONFH immune microenvironment (Figure 7E).

### 3.6 Validation of Hub Gene Expression Levels

**FIGURE 8:**
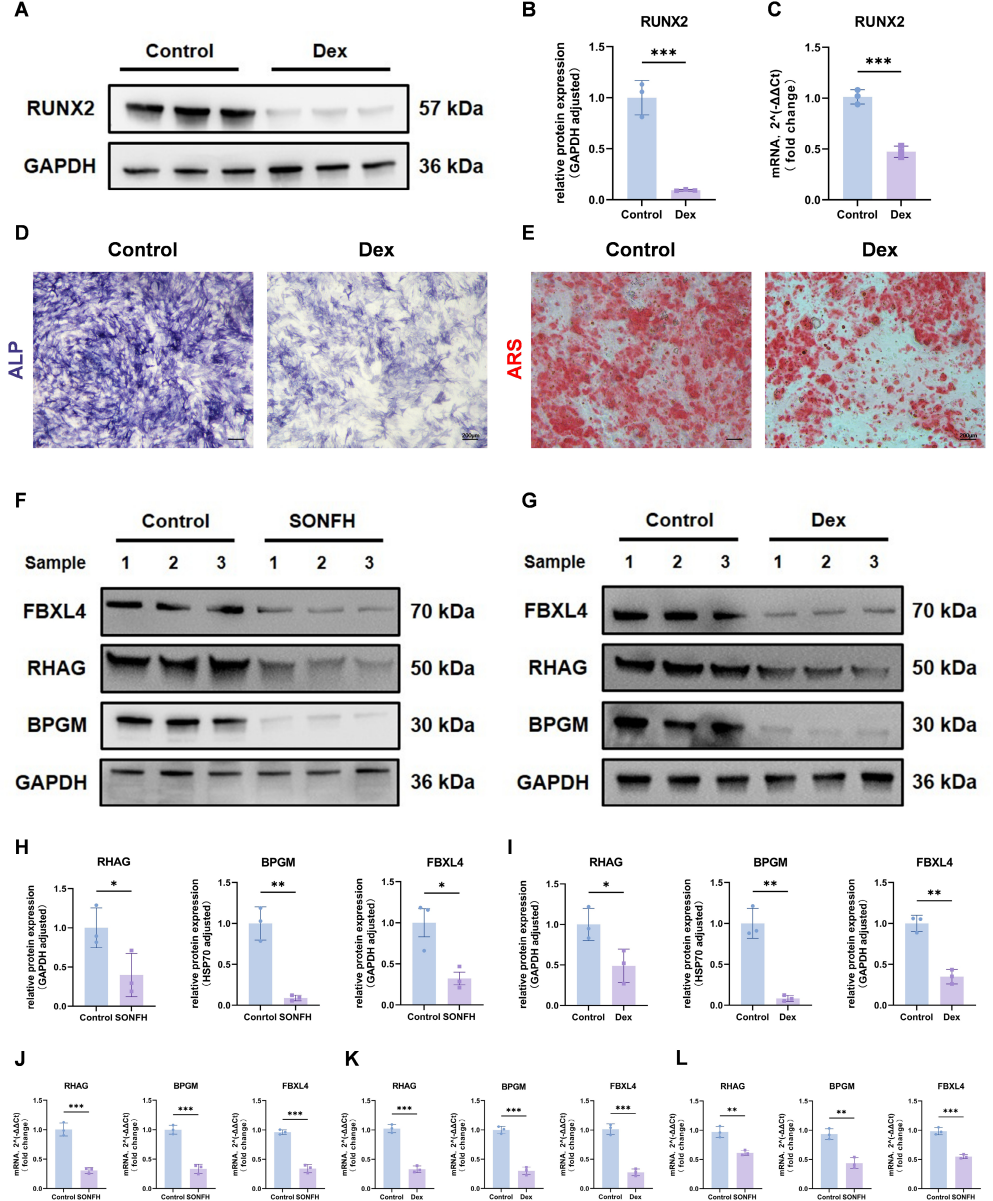
Experimental validation of three key genes. (A, B) Western blot analysis of RUNX2 expression in BMSCs treated with dexamethasone. (C) qRT‐PCR analysis of RUNX2 expression in BMSCs treated with dexamethasone. (D, E) ALP and ARS staining of BMSCs treated with dexamethasone. (F, H, J) Western blot and qRT‐PCR validation of RHAG, BPGM, and FBXL4 expression levels in BMSCs from patients of SONFH and femoral neck fractures. (G, I, G) Western blot and qRT‐PCR validation of RHAG, BPGM, and FBXL4 expression levels in BMSCs following control and dexamethasone treatment. (L) qRT‐PCR validation of RHAG, BPGM, and FBXL4 expression levels in peripheral blood from patients of SONFH and femoral neck fractures.

The above results indicate that BPGM, FBXL4, and RHAG are key lactate‐related genes in SONFH. To experimentally validate these findings, we induced osteogenesis in rat BMSCs cultured under normal conditions and in cells subjected to dexamethasone (Dex) injury. Osteogenic differentiation was first evaluated by assessing the expression of the osteogenesis‐related protein RUNX2 between the two groups. In addition, ALP activity and calcified nodule formation were examined by ALP and alizarin red S (ARS) staining, respectively, to confirm the successful establishment of the injury model. Western blot and qRT‐PCR analyses showed that RUNX2 expression was significantly reduced in Dex‐treated cells compared with controls (Figure 8A–C). Consistent with this, staining results indicated that 10 μM Dex markedly impaired the osteogenic differentiation capacity of BMSCs (Figure 8D,E). We then examined the expression of the three pivotal genes at both the mRNA and protein levels. BPGM, FBXL4, and RHAG were significantly downregulated in Dex‐injured BMSCs (Figure 8C,I,K). Additionally, BMSCs and peripheral blood samples were obtained from patients with avascular necrosis of the femoral head and femoral neck fractures. Following isolation and culture, RNA and protein were extracted and subsequently analyzed by qRT‐PCR and Western blotting. Expression levels of BPGM, FBXL4, and RHAG were significantly reduced in BMSCs from patients with femoral head necrosis (Figure 8F,H,J,L). Collectively, these findings experimentally validate the robustness of our bioinformatics analysis.

## 4 Discussion

Currently, the clinical diagnosis of SONFH primarily relies on imaging examinations, such as MRI, together with characteristic clinical symptoms. However, a substantial proportion of patients already present with irreversible collapse of the femoral head at the time of diagnosis [7]. Moreover, the absence of sensitive and specific early molecular biomarkers severely limits the ability to advance the timing of intervention and to improve subsequent therapeutic outcomes. Previous studies have shown that glucocorticoid‐induced microthrombosis, bone marrow fat deposition, and persistent ischemia markedly impair mitochondrial oxidative phosphorylation in bone tissue [35, 36]. This drives osteocytes and BMSCs to reprogram from oxidative phosphorylation to a highly glycolytic state, resulting in substantial local lactate accumulation [14, 36]. Lactate, beyond being the terminal metabolite of glycolysis, mediates chromatin remodeling, inflammatory responses, and cell fate decisions through histone and non‐histone lactylation [37, 38, 39], suggesting that LRGs may serve as a pivotal metabolic–epigenetic hub linking glucocorticoid exposure to SONFH progression. On this basis, we systematically analyzed transcriptomic data from SONFH patients in the GEO database and, in parallel, screened ligation‐related gene sets in MSigDB to identify LRGs strongly associated with SONFH. We then constructed molecular subtypes and diagnostic models, and subsequently used machine‐learning approaches to identify three core genes—BPGM, FBXL4, and RHAG—that are significantly dysregulated and closely associated with immune infiltration and energy metabolism remodeling. These genes show promise as potential biomarkers of metabolic–immune imbalance in SONFH and may offer new avenues for auxiliary diagnosis and targeted intervention.

Among these core genes, BPGM was initially considered to be expressed predominantly in red blood cells. By catalyzing the production of 2,3‐bisphosphoglycerate (2,3‐BPG), it decreases hemoglobin affinity for oxygen and thereby promotes oxygen release to peripheral tissues under hypoxic conditions [40, 41]. Subsequent studies have shown that BPGM also plays a key regulatory role in cellular metabolic reprogramming by modulating glycolytic intermediates and influencing serine synthesis flux [41]. In addition, BPGM is essential for maintaining glucose metabolism and redox balance in the distal nephron; its deficiency is closely associated with energy metabolism disorders, oxidative stress, and enhanced inflammatory responses [42]. We therefore hypothesize that dysregulation of BPGM may perturb lactate‐mediated epigenetic regulation by altering glycolytic flux and lactate metabolism within the bone marrow microenvironment. This, in turn, could drive a shift in BMSC differentiation from osteogenic to adipogenic lineages, impair osteoblast function, and exacerbate local ischemia–hypoxia and oxidative stress, thereby contributing to the onset and progression of SONFH. FBXL4 is a mitochondria‐localized F‐box protein involved in mitochondrial DNA homeostasis and regulation of oxidative phosphorylation [43]. It contains a characteristic F‐box domain and multiple leucine‐rich repeat (LRR) motifs and functions as a substrate‐recognition subunit of the SCF (SKP1–CUL1–F‐box) E3 ubiquitin ligase complex, mediating ubiquitination and degradation of specific protein substrates. This process governs mitochondrial protein turnover and maintains energy metabolic balance [44]. Biallelic loss‐of‐function mutations in FBXL4 cause mitochondrial DNA deletion/depletion syndromes characterized by mitochondrial dysfunction, elevated lactate levels, progressive neuromuscular disease, and multi‐organ involvement [45, 46], indicating that FBXL4 is critical for preserving mitochondrial oxidative phosphorylation capacity and cellular energy metabolism. Under pathological conditions, aberrant FBXL4 expression or function is thought to promote tissue injury and degenerative changes across multiple organs by disrupting mitochondrial metabolism, aggravating oxidative stress, and enhancing apoptosis [45, 47]. These processes closely mirror the pathophysiology of ischemic necrosis in bone tissue. RHAG was originally identified as a key component of the Rh complex on the erythrocyte membrane [48]. It primarily mediates the transmembrane transport of small molecules such as ammonia and carbon dioxide and contributes to the maintenance of acid–base balance and gas exchange within and outside erythrocytes [49]. Previous studies have shown that RHAG‐deficient erythrocytes exhibit reduced Rh antigen expression on the membrane surface, leading to abnormalities in erythrocyte morphology, cation content, and phospholipid organization [50, 51], changes that may be closely linked to ischemia–hypoxia‐related pathological processes. The expression patterns and biological functions of these three genes in bone metabolism–related diseases, particularly SONFH, have been rarely investigated. In this study, we systematically examined lactate‐related gene expression in SONFH and its association with immune infiltration characteristics, suggesting that these genes may act as potential regulatory factors and diagnostic markers in the development of SONFH.

We performed unsupervised consensus clustering of SONFH patients based on the expression profiles of 20 LR‐DEGs and identified two molecular subtypes, SONFH1 and SONFH2. SONFH2 exhibited overall upregulation of most LRGs, including BPGM, FBXL4, and RHAG, whereas SONFH1 was characterized by high expression of HLA‐DRB1 and TCIRG1. GSVA further revealed increased activity of multiple energy metabolism and substance transport pathways in SONFH2, while SONFH1 was enriched in pathways related to autophagy, stress responses, and inflammatory signaling. These findings indicate marked differences in metabolic status and inflammatory burden between the two subtypes. The synergistic upregulation of lactate‐related genes in SONFH2 may reflect an adaptive response of bone tissue to sustained ischemic stress, enhancing glycolysis and lactate metabolism to maintain local energy supply, modulate transcriptional programs, and partly promote repair and remodeling [52, 53]. This is consistent with the report by Erika et al., which showed that lactate promotes histone lactylation under enhanced glycolysis and increases expression of osteogenic genes such as Runx2 and Sp7 via p300‐mediated mechanisms, thereby facilitating osteoblast differentiation [54]. In contrast, the high expression of immune‐ and resorption‐related genes represented by HLA‐DRB1 and TCIRG1 in SONFH1 suggests enhanced antigen presentation, elevated osteoclast activity, and sustained amplification of inflammatory signaling. This pattern perpetuates a dysregulated bone microenvironment dominated by inflammatory responses and bone resorption, with relatively limited metabolic adaptability [55, 56]. Using three machine‐learning algorithms—SVM‐RFE, LASSO, and RF—for cross‐validation, we identified BPGM, FBXL4, and RHAG as three core LRGs and constructed a nomogram based on these genes to predict SONFH risk. ROC curves, calibration curves, and DCA demonstrated good discrimination, calibration, and clinical net benefit in both the training and validation sets, suggesting that these genes have potential value as auxiliary diagnostic biomarkers and risk‐stratification tools for SONFH. In current clinical practice, SONFH diagnosis relies primarily on imaging and clinical symptoms. However, in the early stages, structural changes of the femoral head are often non‐specific, symptoms are insufficiently specific, and accessible fluid biomarkers are extremely limited, leading to missed opportunities for timely intervention in some patients [57]. Thus, identifying molecular markers that reflect ischemia‐hypoxia, metabolic reprogramming, and bone microenvironment abnormalities is crucial for enabling auxiliary diagnostic and risk stratification in SONFH. Whether these indicators can complement or overcome the limitations of existing diagnostic approaches, however, will require further validation in prospective large‐scale cohort studies and mechanistic functional experiments.

This study, based on CIBERSORT‐derived immune infiltration analysis, revealed significantly increased proportions of memory B cells and activated dendritic cells in SONFH patients, suggesting a distinct state of acquired immune activation within osteonecrotic lesions. Correlation analysis and Mantel tests further demonstrated that expression of BPGM, FBXL4, and RHAG was significantly associated with multiple immune cell infiltration features, including activated dendritic cells, memory B cells, monocytes, M1 macrophages, and activated CD4^+^ T cells. These findings are consistent with previous reports implicating neutrophils, dendritic cells, and B cells in SONFH pathogenesis and further support the concept that SONFH is not merely a focal ischemic necrosis but a systemic bone disease accompanied by complex disturbances in immune homeostasis [58]. Similarly, our observations align with the study by Wang et al., who performed single‐cell RNA sequencing of bone marrow samples from SONFH and femoral neck fracture patients and identified a markedly abnormal neutrophil‐to‐monocyte ratio in SONFH, underscoring the potential role of immune cell imbalance in disease progression [59]. Functionally, increased memory B cells and activated dendritic cells suggest sustained antigen presentation, humoral immune activation, and T‐cell stimulation within SONFH lesions, thereby exacerbating local inflammation and disrupting the balance of bone remodeling [60, 61]. Dendritic cells can indirectly promote osteoclastogenesis and bone resorption by secreting proinflammatory cytokines and modulating the RANKL/OPG axis [62], whereas memory B cells may contribute to bone microenvironment remodeling through antibody production and cytokine networks [63]. The close correlations between BPGM, FBXL4, and RHAG expression and these immune infiltration characteristics suggest that LRGs may occupy key regulatory nodes within the “metabolism–immunity–bone remodeling” axis. We therefore hypothesize that these genes influence the activation state and effector functions of immune cells by modulating local energy metabolism, thereby contributing to the remodeling of the immune microenvironment in SONFH.

To our knowledge, our study is the first bioinformatics analysis to investigate the relationship between SONFH and lactate‐related genes. We identified three core lactate‐related genes—BPGM, RHAG, and FBXL4—as promising biomarkers for the diagnosis and prognosis of steroid‐induced osteonecrosis of the femoral head. However, this study has several limitations. First, although both training and validation datasets were included, the overall sample size remained limited, and the public datasets exhibited substantial heterogeneity. Consequently, both the differential expression results and the machine learning–based screening outcomes may be susceptible to statistical instability. Therefore, these findings require further validation in larger, independent peripheral blood cohorts with consistent sample sources. Second, the expression of the candidate molecules was validated only in human and rat in vitro models, rather than in animal tissue–based models. Accordingly, future work will focus on elucidating the detailed molecular mechanisms of these genes in SONFH‐related lactylation through complementary in vivo and in vitro experiments, thereby laying the groundwork for the development of targeted molecular therapies.

## 5 Conclusion

This study systematically characterized the expression profiles and potential biological functions of LRGs in SONFH using transcriptomic data from the GEO. By integrating differential expression analysis, WGCNA, and screening of MSigDB lactate‐related gene sets, we identified key LRGs significantly associated with SONFH and delineated potential molecular heterogeneity among patients based on their expression patterns. We then applied multiple machine‐learning approaches—including SVM‐RFE, LASSO regression, and RF—to prioritize core genes with diagnostic potential and to construct predictive models, which demonstrated favorable discriminatory performance. In parallel, expression changes of selected candidate genes were preliminarily validated by qRT‐PCR and Western blotting in primary BMSCs derived from rats and humans, as well as in peripheral blood, providing experimental support for the bioinformatics results. Collectively, these findings suggest that LRGs may contribute to SONFH pathogenesis and provide a rationale for future studies aimed at developing adjunctive diagnostic biomarkers and targeted intervention strategies.

## Author Contributions

**Zehua Wang:** writing – original draft, visualization. **Shuhang Dong:** writing – review and editing, visualization. **Kaige Xu:** investigation, software, data curation. **Xinyu Tang:** conceptualization, supervision. **Sijia Guo:** investigation, methodology, formal analysis. **Yaping Jiang:** project administration, writing – review and editing, supervision. **Yingze Zhang:** project administration, supervision, writing – review and editing. **Tao Li:** funding acquisition, project administration, supervision, writing – review and editing.

## Funding

This work was supported by the MOST|National Natural Science Foundation of China (NSFC) (82272489, 82203588), the Taishan Scholar Project of Shandong Province, NO. tsqn202306396, and the 青岛市科学技术局 | Applied Basic Research Fund of Qingdao (青岛市应用基础研究基金项目) (24‐1‐8‐smjk‐3‐nsh).

## Ethics Statement

The animal study protocol was approved by the Ethics Committee of Qingdao University. This study was supported and approved by the Institutional Review Board (IRB) of The Affiliated Hospital of Qingdao University (QYFY WZLL 30495). All clinical sample patients were informed of the purpose of the study and signed the consent form.

## Conflicts of Interest

The authors declare no conflicts of interest.

## Data Availability

The datasets supporting the conclusions of this article are available in the GEO database, https://www.ncbi.nlm.nih.gov/geo/.
